# Influence of Probenecid on the Pharmacokinetics and Pharmacodynamics of Sorafenib

**DOI:** 10.3390/pharmaceutics12090788

**Published:** 2020-08-20

**Authors:** Koen G. A. M. Hussaarts, Leni van Doorn, Karel Eechoute, Jeffrey Damman, Qiang Fu, Nadia van Doorn, Eric D. Eisenmann, Alice A. Gibson, Esther Oomen-de Hoop, Peter de Bruijn, Sharyn D. Baker, Stijn L. W. Koolen, Teun van Gelder, Roelof W. F. van Leeuwen, Ron H. J. Mathijssen, Alex Sparreboom, Sander Bins

**Affiliations:** 1 Department of Medical Oncology, Erasmus MC Cancer Institute, 3015 CE Rotterdam, The Netherlands; g.hussaarts@erasmusmc.nl (K.G.A.M.H.); l.vandoorn@erasmusmc.nl (L.v.D.); k.eechoute@gmail.com (K.E.); n.vandoorn@erasmusmc.nl (N.v.D.); e.oomen-dehoop@erasmusmc.nl (E.O.-d.H.); p.debruijn@erasmusmc.nl (P.d.B.); s.koolen@erasmusmc.nl (S.L.W.K.); r.w.f.vanleeuwen@erasmusmc.nl (R.W.F.v.L.); a.mathijssen@erasmusmc.nl (R.H.J.M.); 2 Department of Pathology, Erasmus MC, 3015 CE Rotterdam, The Netherlands; j.damman@erasmusmc.nl; 3 Division of Pharmaceutics & Pharmaceutical Chemistry, College of Pharmacy & Comprehensive Cancer Center, Ohio State University, Columbus, OH 43210, USA; fu.731@osu.edu (Q.F.); eisenmann.11@osu.edu (E.D.E.); gibson.972@osu.edu (A.A.G.); baker.2480@osu.edu (S.D.B.); sparreboom.1@osu.edu (A.S.); 4 Department of Hospital Pharmacy, Erasmus MC, 3015 CE Rotterdam, The Netherlands; T.van_Gelder1@lumc.nl

**Keywords:** sorafenib, probenecid, hand-foot skin reaction (HFSR), pharmacokinetics, OAT6

## Abstract

Prior studies have demonstrated an organic anion transporter 6 (OAT6)-mediated accumulation of sorafenib in keratinocytes. The OAT6 inhibitor probenecid decreases sorafenib uptake in skin and might, therefore, decrease sorafenib-induced cutaneous adverse events. Here, the influence of probenecid on sorafenib pharmacokinetics and toxicity was investigated. Pharmacokinetic sampling was performed in 16 patients on steady-state sorafenib treatment at days 1 and 15 of the study. Patients received sorafenib (200–800 mg daily) in combination with probenecid (500 mg two times daily (b.i.d.)) on days 2–15. This study was designed to determine bioequivalence with geometric mean Area under the curve from zero to twelve hours (AUC_0–12 h_) as primary endpoint. During concomitant probenecid, sorafenib plasma AUC_0–12 h_ decreased by 27% (90% CI: −38% to −14%; *P* < 0.01). Furthermore, peak and trough levels of sorafenib, as well as sorafenib concentrations in skin, decreased to a similar extent in the presence of probenecid. The metabolic ratio of sorafenib-glucuronide to parent drug increased (+29%) in the presence of probenecid. A decrease in systemic sorafenib concentrations during probenecid administration seems to have influenced cutaneous concentrations. Since sorafenib-glucuronide concentrations increased compared with sorafenib and sorafenib-*N*-oxide, probenecid may have interrupted enterohepatic circulation of sorafenib by inhibition of the organic anion transporting polypeptides 1B1 (OATP1B1). Sorafenib treatment with probenecid is, therefore, not bioequivalent to sorafenib monotherapy. A clear effect of probenecid on sorafenib toxicity could not be identified in this study.

## 1. Introduction

Over the last two decades, systemic anti-cancer treatment options have been expanded from traditional cytotoxic chemotherapy to targeted agents, including tyrosine kinase inhibitors (TKIs). TKIs offer a number of important advantages over conventional cytotoxic chemotherapy like the oral administration of the drugs, but they are still afflicted by some major problems, including large interindividual pharmacokinetic variability, a narrow therapeutic window, and debilitating adverse events [1]. Cutaneous adverse events are among the most frequently observed toxicities with many TKIs, and their intensity can significantly affect both quality of life and health care economics [2]. A particularly painful complication seen most frequently during the early weeks of use with TKIs, such as sorafenib, sunitinib, and regorafenib, is called hand-foot skin reaction (HFSR), in which painful hyperkeratotic plaques develop predominantly over sites of pressure or friction [3,4]. The clinical incidence of HFSR varies among TKIs with a particularly high incidence (20% ≥ grade 3) being observed with sorafenib [5], an orally administered multikinase inhibitor, registered for treatment of advanced hepatocellular carcinoma and advanced renal cell carcinoma as well as iodine-refractory advanced thyroid cancer [3,4,6,7]. Furthermore, it is investigated as a treatment option for acute myeloid leukemia [8]. The pathogenesis of TKI-induced HFSR remains currently unknown, and the only effective treatment options involve either dose reduction or discontinuation of therapy, which theoretically may have negative effects on disease management [9,10]. However, previous in vitro and in vivo research showed that sorafenib can accumulate in human epidermal keratinocytes mediated by the organic anion transporter 6 (OAT6) [11], and that sorafenib-induced skin toxicity can be prevented by cotreatment with the OAT6 inhibitor probenecid without negatively influencing the antitumor properties of sorafenib [11]. Probenecid is an uricosuric agent indicated for the maintenance treatment of hyperuricemia associated with gout and gouty arthritis. It was also used as an adjuvant for therapy with certain antibiotics, such as penicillin, ampicillin, or methicillin, because it elevates and prolongs their plasma levels by inhibition of renal excretion [12]. Probenecid is usually well tolerated at a dose of 500 mg two times daily and is usually taken for (many) months. Probenecid is also known as a pan- uridine diphosphate glucuronosyltransferase (UGT) inhibitor, used in drug registration studies and, therefore, could potentially influence pharmacokinetics of several drugs, including sorafenib that undergoes cytochrome P450 3A4 (CYP3A4) -mediated oxidation into its active metabolite (pyridine-*N*-oxide) and UGT1A9-mediated glucuronidation into sorafenib glucuronide [13,14,15]. Furthermore, probenecid is known to alter the activity of several drug transporters like OAT and the organic anion transporting polypeptides (OATP), which play a main role in renal and hepatic excretion [16]. However, the extent of this possible effect is not yet determined in clinical studies and the safety of the combination of these drugs is currently unknown. As part of an ongoing project to develop translationally useful prevention strategies for sorafenib-induced HFSR, in the current study, we evaluated the pharmacokinetics (PK) and safety of sorafenib when concomitantly used with probenecid.

## 2. Materials and Methods

This non-randomized, cross-over study was performed between November 2017 and November 2019 at the Erasmus University MC Cancer Institute. The study was approved by the local ethics committee of the Erasmus University MC (METC-17-490, date of approval 16-11-2017) and competent authority and was registered at the European Clinical Trials Database (EudraCT 2017-002470-40) and the Dutch trial registry (www.trialregister.nl; number NL6783).

### 2.1. Patients

Patients who had a confirmed diagnosis of advanced hepatocellular carcinoma (HCC) or differentiated thyroid carcinoma with an indication for sorafenib treatment, and who were at least 18 years of age, were included in this study. Furthermore, patients had to have an Eastern Cooperative Oncology Group (ECOG) performance status of ≤2 and an adequate hematological, renal, and liver function defined as a Common Terminology Criteria for Adverse Events (CTCAE) grade of ≤2 at baseline. Besides, patients with known contraindications for probenecid use (e.g., history of uric acid kidney stones, an acute gouty attack, or blood dyscrasias) and/or the use of drugs that are strong CYP3A4 or UGT1A9 inducers or inhibitors were excluded. All included patients gave written informed consent.

### 2.2. Study Procedures

Patients received sorafenib for at least two weeks to ensure steady-state pharmacokinetics of sorafenib. Since sorafenib has linear pharmacokinetics, [17] dose reductions were allowed after the start of the study. Sorafenib was administered at a 200–800-mg daily dose during the 15-day study period and was given concomitantly with probenecid (500 mg b.i.d.) from day 2 to day 15 of the study. Both sorafenib and probenecid were ingested at predefined timepoints at 10:00 a.m. and 10:00 p.m.

### 2.3. Pharmacokinetic Sampling

Patients were admitted to the hospital on days 1 and 15 of the study for pharmacokinetic blood sampling. A total of nine blood samples for the determination of sorafenib, sorafenib *N*-oxide, and sorafenib glucuronide were obtained at predefined time points (T = pre, T = 0.5 h, T = 1 h, T = 2 h, T = 4 h, T = 6 h, T = 8 h, T = 10 h, and T = 12 h). Blood samples were processed into plasma within 30 min, by vortex mixing and centrifugation for 10 min at 2500× *g* at 4 °C. Plasma concentrations were determined using a validated liquid chromatography tandem mass spectrometry (LC-MS/MS) method [18], at both the laboratory of Translational Pharmacology in the Erasmus MC, Rotterdam, and the laboratory of Pharmaceutics and Pharmaceutical Chemistry, Ohio State University, OH. Predefined pharmacokinetic endpoints were the dose-corrected area under the curve from preadministration time point until 12 h after sorafenib intake (AUC_0–12 h_), maximum concentration (C_max_), time until maximum concentration (T_max_), and lowest plasma concentration (C_trough_) and were determined using WinNonlin v. 7.0 (Phoenix, Certara, 5349 AB, Oss, The Netherlands) for sorafenib, sorafenib-*N*-oxide, and sorafenib glucuronide.

### 2.4. Skin Biopsies

A 3-mm skin biopsy was obtained at days 1 and 15 of the study during PK sampling days for pharmacokinetic analysis. Skin biopsies were taken from either the forearm or the shoulder region, but always from the same region at the same timepoint in an individual patient during the two consecutive PK sampling days. If patients had HFSR lesions at the hand at the first PK day, an additional skin biopsy was performed from the thenar eminence region of the hand for pathologic analysis on days 1 and 15. The biopsies were graded according to the scoring for interface dermatitis as used for graft-versus-host disease by an experienced pathologist (J.D.). There is no other pathologic grading scale for HFSR and our grading scale shows the most overlapping features from a pathologic perspective [19]. Furthermore, concentrations of sorafenib were determined from the skin biopsies after dilution in human plasma and homogenization using the validated liquid chromatography tandem mass spectrometry (LC-MS/MS) method described earlier [18].

### 2.5. In Vitro Transport Assay

Transport assays assessing probenecid’s inhibition of OATP1B1 were conducted as previously described [20]. The [3H]estradiol-17b-d-glucuronide, a positive control substrate for OATP1B1 [21], was obtained from American Radiolabeled Chemicals. Water-soluble probenecid was obtained from Invitrogen (Molecular Probes). The generation and characterization of Flp-In T-Rex293 cells expressing inducible OATP1B1 have been reported previously [22,23]. Cells expressing OATP1B1 or vector control (VC) were cultured in Dulbecco’s Modified Eagle’s Medium (DMEM; Invitrogen) supplemented with 10% Fetal Bovine Serum (FBS), hygromycin B (25 mg/mL; Invitrogen), and blasticidin (37.5 mg/mL; Biovision, CA, USA).

Cells were seeded in 24-well plates in phenol red-free DMEM containing 10% FBS, hygromycin (25 mg/mL), blasticidin (37.5 mg/mL), and doxycycline (1 µg/mL) and were incubated at 37 °C for 24 h. Cells were then washed with warm phosphate buffered saline PBS and pre-incubated with the indicated concentration of probenecid in phenol red-free DMEM (without FBS and supplements) at 37 °C for 15 min. Cells were then incubated with phenol red-free DMEM containing the indicated concentration of probenecid and 0.2 µM [3H]estradiol-17b-d-glucuronide for an additional 15 min. The experiment was terminated by washing three times with ice-cold PBS. Cells were lysed in 1 N NaOH at 4 °C overnight, and then the solution was neutralized with 2 mol/L HCl. Total protein was measured using a Pierce BCA Protein Assay Kit (Thermo Scientific), and total protein content was quantified using a microplate spectrophotometer. Drug concentrations were determined in the remaining cell lysate by liquid scintillation counting using a scintillation counter. OATP1B1-mediated uptake was calculated by dividing the disintegrations per minute (dpm) from each replicate by the amount of protein (mg) and subtracting the dpm/mg protein in 2 cell line from the dpm/mg protein in OATP1B1 overexpressing cells at each concentration of probenecid. OATP1B1-mediated uptake at each concentration of probenecid was then compared with OATP1B1-mediated uptake when only an equal volume of vehicle was added without probenecid (i.e., % control). The half maximal inhibitory concentration (IC_50_) was calculated using a nonlinear fit comparing concentration of probenecid versus response.

### 2.6. Toxicity

Toxicity rates were determined at baseline, days 1 and day 15 of the study using Common Terminology Criteria for Adverse Events (CTCAE version 4.0, National Cancer Institute, Bethesda, MD, USA), and by evaluating the patient diaries during the sorafenib monotherapy phase and sorafenib concomitant with probenecid phase.

### 2.7. Statistical Analysis

The primary objective of this study was to determine bioequivalence between sorafenib monotherapy and sorafenib concomitantly with probenecid to determine whether it is a safe option in clinical practice. Bioequivalence can be concluded when the 90% confidence interval (CI) of the ratio of geometric means is within 80% and 125%. Assuming a standard deviation of the difference of 0.25 for log(AUCsorafenib), using a 90% power and two-sided alpha of 5%, the required number of evaluable patients was 16. Analyses of AUC_0–12 h_, C_trough_, and C_max_ were performed on log-transformed observations by means of the paired t-test. The point estimates and CIs were transformed back to the original scale in order to give the point estimates for the ratio of the geometric means and the CIs. T_max_ was analyzed by means of the Wilcoxon signed rank test and described with medians and interquartile ranges. Toxicity was described as the incidence of toxicity per phase. This was corrected for baseline toxicity and was only taken into account in case of an increase in CTCAE grade per PK sampling day. Since the design of this study was not appropriate to detect a significant difference in toxicity, these results had a descriptive character.

## 3. Results

### 3.1. Patient Characteristics

Seventeen patients were included, of whom 16 patients were evaluable due to withdrawal of one patient. Most patients (n = 14) were male and had an HCC (n = 12). Eight patients with HCC had underlying liver cirrhosis due to alcohol abuse (n = 3) or chronic viral hepatitis (n = 5). Other patient characteristics can be found in Table 1.

### 3.2. Pharmacokinetics

**Figure 1:**
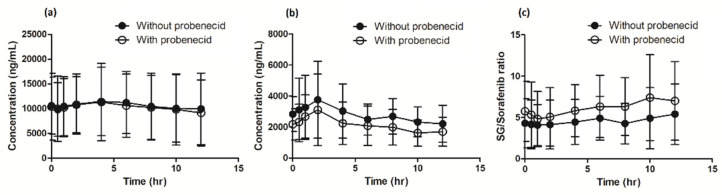
Pharmacokinetic results are displayed for (**a**) sorafenib-glucuronide concentration, (**b**) sorafenib concentration, (**c**) sorafenib-glucuronide (SG) to sorafenib ratio.

When sorafenib was administered with concomitant probenecid, the geometric mean sorafenib AUC_0–12 h_ was 26.8% (90% CI: −37.7% to −14.1%) lower than when sorafenib was administered alone. Similarly, sorafenib plasma C_max_ and C_trough_ decreased significantly by 25.1% (90% CI: −44.3% to −19.7%) and 26.0% (90% CI: −43.4% to −3.4%), respectively (Table 2 and Figure 1). Sorafenib metabolites showed a similar decrease in plasma concentration, although there was a substantial interpatient variability. The sorafenib-*N*-Oxide AUC_0–12 h_ decreased by 36.3% (90% CI: −52.8% to −14.1%) and C_max_ showed a similar significant decrease of 39.2% (90% CI: −54.6% to −18.7%). Interestingly, cotreatment with probenecid did not decrease the sorafenib-glucuronide AUC_0–12 h_ to a similar extent (5.5%; 90% CI: −18.0% to 8.9%), did not significantly influence C_max_ (6.1%; 90% CI: −21.7% to 12.7%), and, thus, shows bioequivalence (Table 2 and Figure 1). The ratio of sorafenib-glucuronide to sorafenib increased by 29% when sorafenib was co-administered with probenecid, whereas other metabolic ratios did not change significantly. Sorafenib concomitant with probenecid is not bioequivalent to sorafenib monotherapy.

Sorafenib concentration in skin decreased in the presence of probenecid by 28.1% (90% CI: −46.3% to −3.7%), with a similar plasma sorafenib/sorafenib in skin ratio (Table 2). Furthermore, there was no difference between patients with or without liver cirrhosis in sorafenib plasma AUC (−6.3%; 90% CI: −32.9% to 30.7%; *P* = 0.73) and C_trough_ (−7.4%; 90% CI: −46.8% to 61.3%; *P* = 0.81).

### 3.3. In Vitro Transport Assay

**Figure 2:**
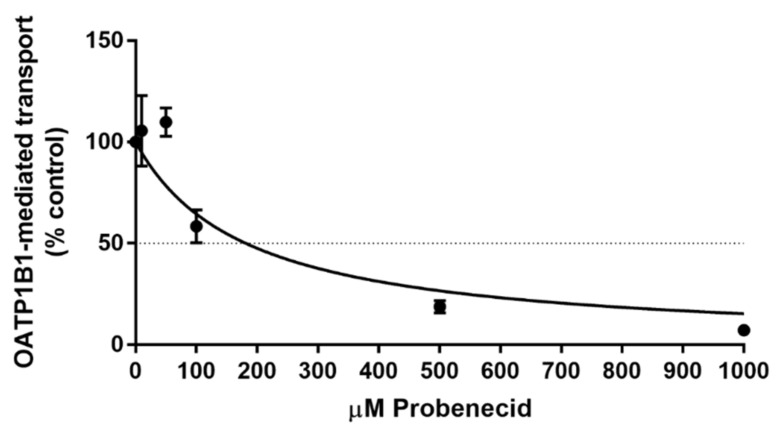
Inhibition of OATP1B1 function by probenecid in vitro. HEK293 cells expressing OATP1B1 or VC were pre-incubated with probenecid at the indicated concentrations for 15 min before incubation with probenecid and [3H]estradiol-17b-d-glucuronide for 15 min. Data represent uptake of OATP1B1-expressing cells at each concentration compared against vehicle after subtracting uptake by VC cells (mean ± SEM). Each concentration consists of 3–9 technical replicates across 1–3 biological replicates.

Subsequently, we hypothesized that probenecid interferes with enterohepatic sorafenib circulation via OATP1B1 inhibition and, therefore, measured the impact of probenecid on the cellular uptake of a probe OATP1B1 substrate, [3H]estradiol-17b-d-glucuronide, in a cell line overexpressing OATP1B1. Probenecid inhibited OATP1B1 function with an IC_50_ of 182 µM (Figure 2). Given that probenecid achieves plasma concentrations higher than 200 µM at clinically relevant doses, [24] the results of this experiment support our hypothesis that OATP1B1 contributes to the observed drug–drug interaction between probenecid and sorafenib.

### 3.4. Toxicity

There were three serious adverse events, which were assumed to be not related to any of the study drugs (gastroenteritis with dehydration and dyspnea grade 3 during the probenecid part and atrial fibrillation de novo in the monotherapy part, all complicated with unplanned hospitalization). HFSR, rash, anorexia, and fatigue occurred more frequently during probenecid administration (65%) than during sorafenib monotherapy (43%) (Table 3, Supplementary Table S1). HFSR occurred in 10 of 16 patients with five patients experiencing HFSR at the first day of PK sampling. Three of these patients experienced progression of HFSR symptoms during the study. Most toxicity occurred after 3–6 weeks of treatment. Most grades 2 and 3 adverse events were seen when sorafenib was administered with probenecid. A total of five patients experienced HFSR at PK sampling day 1 and a biopsy of the thenar eminence region was taken in these patients on days 1 and 15 of the study. There was no difference in the grading of the HFSR between the first and second PK sampling day in these patients.

## 4. Discussion

In this study, we investigated the influence of the OAT6 and OATP1B1 inhibitor probenecid on sorafenib pharmacokinetics and toxicity in patients, and found a significant decrease in the geometric mean of sorafenib plasma exposure and a nearly significant decrease in intracutaneous sorafenib exposure during concomitant probenecid administration making sorafenib concomitantly with probenecid not bioequivalent to sorafenib monotherapy. Of the metabolites, systemic sorafenib-*N*-oxide concentrations decreased proportionally with the parent drug, but the sorafenib-glucuronide to sorafenib ratio increased after probenecid administration, which does not support the hypothesis of UGT inhibition. This is in line with our previous findings on enterohepatic circulation of sorafenib-glucuronide, which demonstrated that OATP1B inhibition leads to an increase in plasma sorafenib glucuronide levels [13,25]. Next to the relative increase in systemic sorafenib-glucuronide exposure, its reduced hepatocellular secretion would also explain the decrease in systemic sorafenib concentrations after probenecid administration, as these concentrations are less maintained via enterohepatic circulation of deconjugated sorafenib glucuronide. Moreover, as we found probenecid to inhibit OATP1B1-mediated transport in vitro at clinically relevant concentrations and as we previously showed that OATP1B1 contributes to enterohepatic sorafenib cycling [13], it is plausible that reduced enterohepatic circulation of sorafenib led to its significant decrease in systemic exposure after probenecid. Alternatively, the relative systemic accumulation of sorafenib-glucuronide compared with sorafenib and sorafenib-*N*-oxide might be caused by decreased tubular secretion in the kidney, where probenecid is known to inhibit prominent drug transporters as OAT1 and OAT3 [16]. However, data regarding this potential interaction are lacking. This study was not designed to quantify these mechanisms and it should be noted that all patients followed the same sequence of treatment, i.e., sorafenib monotherapy followed by concomitant probenecid, which complicates the differentiation between effects of probenecid and time. Regardless of its etiology, the decrease in systemic sorafenib exposure rather than inhibited OAT6-mediated transport seemed to determine cutaneous sorafenib concentrations, as systemic and cutaneous sorafenib concentrations decreased proportionally and protective effect of probenecid on cutaneous exposure could not be demonstrated. This follows a recent population PK analysis in which systemic sorafenib and sorafenib-*N*-oxide were associated with earlier occurrence of HFSR [26]. Despite lower sorafenib exposure, adverse events occurred more frequently during probenecid cotreatment. It is known that the prevalence of adverse events increases during the first weeks of sorafenib treatment [27]. The difference in adverse events is, therefore, unlikely a result of the drug interaction observed in this study. Patients participated in the study at a relatively early stage of the TKI treatment (i.e., maximal six weeks after start of sorafenib treatment). Usually sorafenib adverse events such as hypertension occur early during TKI treatment [27] and HFSR usually develops 2–4 weeks after initiation of sorafenib. Hence, it is not likely that we missed this adverse event in our study population [19,27]. In the five patients who experienced HFSR at the first day of study, pathologic characteristics of skin biopsies from the thenar eminence region did not change during the study, potentially due to the non-specificity for HFSR of the used grading scale, i.e., the interface dermatitis score, or due to the absence of high-grade HFSR in our study population. Therefore, subtle HFSR specific changes could have been missed.

## 5. Conclusions

In conclusion, both systemic and cutaneous sorafenib exposure decreased proportionally during concomitant probenecid administration, which may have been caused by interruption of enterohepatic cycling via OATP1B1 inhibition.

## Figures and Tables

**Table 1:** Patient characteristics.

Characteristic	Evaluable Patients (n = 16)
Sex	
Male	14 (88%)
Female	2 (12%)
Age (years) median [IQR]	66.5 (58–75)
Performance	
ECOG 0	1 (6%)
ECOG 1	13 (82%)
ECOG 2	2 (12%)
Tumor type	
HCC	12 (72%)
• Liver cirrhosis	8 (66%)
• Pre-existent hepatitis	5 (42%)
Thyroid carcinoma	4 (28%)
BMI (kg/m^2^) median [IQR]	25.2 (22–30)
Race	
Caucasian	11 (70%)
African	1 (6%)
Arabic	3 (18%)
Asian	1 (6%)
Sorafenib daily dose at start of study	
200 mg	1 (6%)
400 mg	10 (63%)
600 mg	2 (12%)
800 mg	3 (19%)

Abbreviations: BMI = body mass index, ECOG = Eastern Cooperative Oncology Group; HCC = hepatocellular carcinoma, n = number of patients; IQR = interquartile range.

**Table 2:** Pharmacokinetic results.

Pharmacokinetic Parameters	Sorafenib Monotherapy	Metabolic Ratio (Metabolite/Sorafenib)	Sorafenib with Probenecid	Metabolic Ratio (Metabolite/Sorafenib)	RD Sorafenib Monotherapy vs. Sorafenib + Probenecid (90% CI)	
**Sorafenib**					
AUC_0–12 h_ (CV%)	33,457.8 (42)		24,476.8 (57)		−26.8% (−37.7% to −14.1%)	
geomean μg·h/mL						
C_max_ (CV%)	4457.8 (52)		3337.2 (63)		−25.1% (−44.3% to −19.7%)	
geomean μg/mL						
C_trough_ (CV%)	2125.5 (60)		1571.9 (61)		−26.0% (−43.4% to −3.4%)	
geomean μg/mL						
T_max_ (IQR)	3.7 (1.5–4.15)		2.2 (1.0–2.01)		NA	
median hours						
**Sorafenib-*N*-oxide**					
AUC_0–12 h_ (CV%)	3442.8 (78)	0.10	2192.3 (77)	0.09	−36.3% (−52.8% to −14.1%)	
geomean μg·h/mL						
C_max_ (CV%)	467.3 (77)		283.9 (74)		−39.2% (−54.6% to −18.7%)	
geomean μg/mL						
C_trough_ (CV%)	271.1 (282)		(71)		−35.2% (−59.7% to 4.3%)	
geomean μg/mL						
**Sorafenib-glucuronide**					
AUC_0–12 h_ (CV%)	120,660 (55)	3.61	113,995 (59)	4.66	−5.5% (−18.0% to 8.9%)	
geomean μg·h/mL						
C_max_ (CV%)	12,704 (51)		11,931 (64)		−6.1% (−21.7% to 12.7%)	
geomean μg/mL						
C_trough_ (CV%)	9159 (65)		8400 (67)		−8.3% (−21.3% to 6.9%)	
geomean μg/mL						
**Tissue**					
Sorafenib concentration in keratinocytes	50.0 (61)	1.49 × 10^−3 #^	36.0 (63)	1.47 × 10^−3^	−28.1% (−46.3% to −3.7%)	
Geomean ng/mL (CV%)						

Abbreviations: AUC_0–12 h_ = area under the curve, time point 0 h to 12 h; CI = confidence interval; RD = relative difference; C_max_ = maximum concentration; C_trough_ = minimum concentration; CV = coefficient of variation; h = hours; n = number of patients; T_max_ = time until maximum concentration, IQR = interquartile range; NA = not applicable. ^#^ = ratio of plasma to skin sorafenib.

**Table 3:** Patient reported adverse events during study period.

	Sorafenib Mono (N = 16)		Sorafenib Concomitantly with Probenecid (N = 16)	
Adverse event	Grade 1–2	Grade 3	Grade 1–2	Grade 3
HFSR	3	-	6	1
Rash	1	-	3	1
Nausea	1	-	2	-
Vomiting	0	-	1	-
Oral mucositis	1	-	1	-
Diarrhea	1	-	2	-
Constipation	2	-	3	-
Anorexia	4	-	7	-
Dyspnea	-	-	-	1
Edema	-	-	1	-
Fatigue	2	-	6	1
Fever	1	-	-	-
Pain	1	1	2	1
Serious adverse events (SAE)	1		2	

There were three serious adverse events (atrial fibrillation de novo, dyspnea grade 3, and severe gastroenteritis with dehydration) during sorafenib therapy for which hospitalization was necessary.

## Supplementary Materials

The following are available online at https://www.mdpi.com/1999-4923/12/9/788/s1, Table S1: Toxicity per patient.
