# Supplementary material for: Influence of Probenecid on the Pharmacokinetics and Pharmacodynamics of Sorafenib
Source: Pharmaceutics. 2020 Aug 20;12(9):788. doi: 10.3390/pharmaceutics12090788 (PMC7559746; doi:10.3390/pharmaceutics12090788)
Supplement: Supplementary file 1 [file pharmaceutics-12-00788-s001.pdf]

pharmaceutics

Supplementary Materials: Influence of Probenecid on the Pharmacokinetics and Pharmacodynamics of Sorafenib

Koen G. A. M. Hussaarts, Leni van Doorn, Karel Eechoute, Jeffrey Damman, Qiang Fu, Nadia van Doorn, Eric D. Eisenmann, Alice A. Gibson, Esther Oomen-de Hoop, Peter de Bruijn, Sharyn D. Baker, Stijn L. W. Koolen, Teun van Gelder, Roelof W. F. van Leeuwen, Ron H. J. Mathijssen, Alex Sparreboom and Sander Bins

Table S1. toxicity per patient.

Patient	Duration of sorafenib at baseline (days)	Sorafenib daily dose (mg)	Study phase	HFSR	Rash	Nausea	Diarrhea	Constipation	Anorexia	Fatigue	Pain	Hoarseness
			Baseline					1	1	1	1	
**1**	14	800	PK day 1		2			1		1	1	
		800	PK day 2					1	1	1	1	
			Baseline			1			1		1	
**2**	28	400	PK day 1							1	1	
		400	PK day 2			1			1	1	1	
			Baseline						1	1	1	
**3**	9	400	PK day 1		1				2	2	1	
		400	PK day 2						1	1	1	
			Baseline							1	1	
**4**	27	600	PK day 1				1		1	1	1	
		600	PK day 2	1					2	2	1	
			Baseline					1	1	1		
**5**	23	800	PK day 1	1				1	1	1	1	1
		800	PK day 2					2	1	1	1	
			Baseline									
**6**	35	800	PK day 1			1				1	1	
		800	PK day 2	3	2					1	2	
			Baseline	1				1		1	1	1
**7**	22	400	PK day 1	1				1		1	1	
		400	PK day 2	2				1		1	1	
			Baseline							1	1	1
**8**	8	400	PK day 1							1	1	
		600	PK day 2	1					1	1	1	
			Baseline						1	1		
**9**	11	400	PK day 1					1	1	1		
		400	PK day 2	1	1			1	1	1		
			Baseline						1	1	1	1
**10**	23	200	PK day 1	1					1	1	1	
		200	PK day 2			1			1	2	1	1
			Baseline		1						1	1
**11**	42	400	PK day 1							2	1	
		400	PK day 2				1			2		
			Baseline		1							1
**12**	12	400	PK day 1									
		400	PK day 2	2	1					1		
			Baseline	1						1	1	1
**13**	41	400	PK day 1	1					1	1	3	
		400	PK day 2	2				1	1	1	3	1
			Baseline	1						2		1
**14**	29	400	PK day 1	1					1	2		1
		400	PK day 2		3				2	3	1	1
			Baseline		1		1		1			
**15**	24	400	PK day 1				1		1	1		
		400	PK day 2				1		1	1		
			Baseline						1	2		
**16**	23	400	PK day 1						1	2		
		400	PK day 2				2		2	2		

Legend: Toxicity per phase per patient according to the CTCAE grading. In general there is an increase in adverse events during the study. Fields are left blank if toxicity is scored 0.
